# Highly conserved regions in Ebola virus RNA dependent RNA polymerase may be act as a universal novel peptide vaccine target: a computational approach

**DOI:** 10.1186/s40203-015-0011-4

**Published:** 2015-08-08

**Authors:** Arafat Rahman Oany, Tahmina Sharmin, Afrin Sultana Chowdhury, Tahmina Pervin Jyoti, Md. Anayet Hasan

**Affiliations:** Department of Biotechnology and Genetic Engineering, Faculty of Life Science, Mawlana Bhashani Science and Technology University, Santosh, Tangail-1902, Bangladesh; Department of Genetic Engineering and Biotechnology, Faculty of Biological Sciences, University of Chittagong, Chittagong-4331, Bangladesh; Biotechnology and Genetic Engineering Discipline, Life Science School, Khulna University, Khulna-9208, Bangladesh

**Keywords:** Ebola, Computational approach, RNA polymerase, Epitope, Vaccine

## Abstract

**Purpose:**

Ebola virus (EBOV) is such kind of virus which is responsible for 23,825 cases and 9675 deaths worldwide only in 2014 and with an average diseases fatality rate between 25 % and 90 %. Although, medical technology has tried to handle the problems, there is no Food and Drug Administration (FDA)-approved therapeutics or vaccines available for the prevention, post exposure, or treatment of Ebola virus disease (EVD).

**Methods:**

In the present study, we used the immunoinformatics approach to design a potential epitope-based vaccine against the RNA-dependent RNA polymerase-L of EBOV. BioEdit v7.2.3 sequence alignment editor, Jalview v2 and CLC Sequence Viewer v7.0.2 were used for the initial sequence analysis for securing the conservancy from the sequences. Later the Immune Epitope Database and Analysis Resource (IEDB-AR) was used for the identification of T-cell and B-cellepitopes associated with type I and II major histocompatibility complex molecules analysis. Finally, the population coverage analysis was employed.

**Results:**

The core epitope “FRYEFTAPF” was found to be the most potential one, with 100 % conservancy among all the strains of EBOV. It also interacted with both type I and II major histocompatibility complex molecules and is considered as nonallergenic in nature. Finally, with impressive cumulative population coverage of 99.87 % for the both MHC-I and MHC-II class throughout the world population was found for the proposed epitope.

**Conclusion:**

To end, the projected peptide gave us a solid stand to propose for vaccine consideration and that might be experimented for its potency in eliciting immunity through humoral and cell mediated immune responses *in vitro* and *in vivo*.

**Electronic supplementary material:**

The online version of this article (doi:10.1186/s40203-015-0011-4) contains supplementary material, which is available to authorized users.

## Background

EVD, previously designated as Ebola haemorrhagic fever, is a fatal disease in humans and other mammals (monkeys, chimpanzees and gorillas) (Choi and Croyle 2013, Leroy et al. 2004, Sullivan et al. 2000). The fatality rate of EDV is varied from 25 to 90 % with an average of about 50 % (Peters and Peters 1999) and it is caused by a virus of the family Filoviridae, genus Ebolavirus. There are five separate Ebola virus species have been identified, four of which are disease causing to humans: Ebola virus (Zaire ebolavirus); Taï Forest virus (Taï Forest ebolavirus, formerly Côte d’Ivoire ebolavirus); Sudan virus (Sudan ebolavirus); and Bundibugyo virus (Bundibugyoebolavirus) (Hoenen et al. 2012). The fifth one, Reston virus (Reston ebolavirus), is harmful to nonhuman primates, but not to humans (Elisha and Adegboro 2014, Geisbert et al. 2009). Among the recognized species of ebolavirus, the notoriously deadly Zaire ebolavirus is responsible for epidemics which have been taken place mainly in African countries including Democratic Republic of Congo, Uganda, Sudan, the Ivory Coast, and Gabon (Baize et al. 2014, Chowell et al. 2004, Feldmann et al. 2003, Frieden et al. 2014, Hewlett and Hewlett 2005, Kuhn et al. 2010, Li and Chen 2014, Rouquet et al. 2005). This virus is passed on people from wild animals and through human-to-human contact transmits in the human population. Those are infected with this virus bear fearsome symptoms, including high fever, hemoptysis, impaired kidney and liver function and severe internal bleeding (Gatherer 2014, Goeijenbier et al. 2014, Keiser et al. 2004, Peters and Peters 1999). In the fall of 2014 the Ebola virus gained widespread attention when in West Africa the largest outbreak has been reported in history.

The EBOV genome is a single-stranded, negative-sense, non-segmented RNA approximately 19 kb long. It codes for seven tandemly arranged viral genes which order is 3′ leader- NP (nucleoprotein) - VP35 (virion protein 35)-VP40- GP (glycoprotein)-VP30-VP24- L (RNA-dependent RNA polymerase)-trailer −5′. Transcription and translation of this viral genome result in the synthesis of seven structural proteins and a single non-structural, secreted glycoprotein (Feldmann et al. 1999). Three of the structural proteins are membrane-associated proteins; GP is a type I transmembrane protein, while VP24 and VP40 are placed on the inner surface of the membrane. The remaining four, NP, VP30 (transcription factor), VP35 (polymerase cofactor), and L (RNA-dependent RNA polymerase), are essential to viral genomic RNA to form the ribonucleoprotein complex. These proteins have been shown to be necessary and sufficient for EBOV transcription and replication (Crary et al. 2003, Feldmann et al. 2001, Mühlberger et al. 1998; 1999, Takada et al. 1997).

To date, information regarding the processing, structure and functions of Ebola virus (EBOV) protein L (EBOL) demonstrates that it is an RNA-dependent RNA polymerase, with the assistance of VP35. It also shows mRNA (guanine-N (7)-)-methyltransferase, mRNA guanylyltransferase and poly (A) synthetase activities which are essential for the replication and transcription of EBOV (Poch et al. 1990). The viral mRNA guanylyltransferase serves either as transcriptase or as replicase. The transcriptase synthesizes subgenomic RNAs, assures their capping and polyadenylation. The transcriptase stutters on a specific sequence, leads to a co-transcriptional editing of the glycoprotein (GP) mRNA. In replicase mode, the polymerase replicates the viral genome without recognizing the transcriptional signals. These reports suggest that EBOL is an important cellular component for the transcription and replication of the EBOV genome and, as such, plays a key role in the EBOV life cycle.

Due to the emergence of Ebola virus outbreak, there is an immediate need to determine novel therapeutic targets against this pathogen. The identification of specific epitopes derived from infectious pathogens has significantly advanced the development of epitope-based vaccines (EVs). Bettered understanding of the molecular basis of antigen recognition and HLA binding motifs has resulted in the advancement of rationally designed vaccines depend on algorithms predicting the peptide’s binding to human HLA. In comparison to the conventional vaccines, peptide or epitope based vaccines are easy to develop, chemical stable, more specific, and free of any infectious or oncogenic potential hazard (Holland and Domingo 1998, Sette et al. 2002). Though EVs have varied advantages, the wet lab based discovery of candidate epitopes is expensive and time consuming. Furthermore, for the final selection of epitopes various immunological requirements are needed to be considered. As a result computational methods, an alternative *in silico* approaches (Germain 1994) have recently been attracting growing interest of the researchers for predicting epitopes with reduced cost and time. The application of bioinformatics in immunology is termed as immunoinformatics. Currently, numerous immunoinformatics tools are available for identifying B and T cell epitopes and human leukocyte antigen (HLA) ligands (Petrovsky and Brusic 2002, Poland et al. 2009, Sette and Fikes 2003) with high sensitivity and specificity. The ‘immunoinformatics’ approach has already proven its potency in the case of human immunodeficiency virus (Wilson et al. 2003), multiple sclerosis (Bourdette et al. 2005), tuberculosis (Robinson and Amara 2005) and malaria (López et al. 2001) with desired results. In the present study, we have followed immunoinformatics approaches for designing potential conserved epitope candidate for the utility of vaccine development against the deadly Ebola virus, with an expectation of further wet lab validation.

## Methods

### Sequence retrieval and conserved region identification

The protein sequences of the RNA-dependent RNA polymerase-L (Volchkov et al. 1999) of the EBOV were retrieved from the UniProtKB (Apweiler et al. 2004) database in the FASTA format. BioEdit v7.2.3 sequence alignment editor (Apweiler et al. 2004) was used for the identification of the conserved region among the sequences through multiple-sequence alignment (MSA) with ClustalW (Hall 1999). Finally, Jalview v2 tool (Thompson et al. 1994) was used to retrieve the alignment and the CLC Sequence Viewer v7.0.2 (http://www.clcbio.com) was used for analysis of the divergence among the different strains of the EBOV.

### Antigenicity determination of the conserved peptides

VaxiJen v2. 0, a Web-based server (Waterhouse et al. 2009, Doytchinova and Flower 2007) was used for the determination of the antigenicity of the conserved sequences. Herein, we used the default parameters for the prediction, with a threshold value of 0.4.

### T-cell epitope prediction

For this study, two online servers were used. Firstly, the NetCTL v1.2 server (Larsen et al. 2007) was used for predicting potential cytotoxic T lymphocyte (CTL) epitopes from the conserved peptides. Here for predicting the epitopes, we used a combined algorithm including major histocompatibility complex class I (MHC-I) binding, transporter of antigenic peptide (TAP) transport efficiency, and proteasomal C terminal cleavage prediction. Depending on the score, the best candidates were picked for further investigation. The epitope prediction was confined to 12 MHC-I supertypes. MHC-I binding and proteasomal cleavage were carried out through artificial neural networks and the weight matrix was used to estimate the TAP transport efficiency. The threshold value for epitope identification was set at 0.5 for maintaining sensitivity and specificity of 0.89 and 0.94, respectively during the analysis. This would support to assess the findings more decisively by developing more epitopes. Finally, for confirming the prediction with default parameters, CTLPred (Bhasin and Raghava 2004) was employed additionally.

### MHC-I and MHC-II restriction analysis

Furthermore, from the Immune Epitope Database and Analysis Resource (IEDB-AR), T Cell Epitope Prediction Tools was implied for the identification of MHC-I (Hoof et al. 2009, Nielsen et al. 2007) and MHC-II (Wang et al. 2008; 2010) binding of the peptide. In order to calculate the half-maximal inhibitory concentration (IC_50_) values required for peptide binding to MHC-I molecules, Stabilized Matrix Method (Peters and Sette 2005) was applied with a preset 9.0-mer epitope.

In case of MHC-II binding analysis, the IEDB-recommended method was used for the specific HLA-DQ, HLA-DP, and HLA-DR loci. Herein, specific peptides were used to predict the MHC-II interaction on the basis of MHC-I analysis and antigenic conservancy.

### Prediction of population coverage

Population coverage for epitope was assessed by the IEDB population coverage calculation tool (Bui et al. 2006). Here we used the allelic frequency of the interacting HLA alleles for the prediction of the population coverage for the corresponding epitope.

### B-cell epitope prediction

Linear B cell epitopes are of different lengths of peptides from 2 to 85 in comparison to that of T cell epitopes. B-cell epitope produces immune response when it interacts with B lymphocytes. It then initiates the differentiation of B lymphocytes into plasma and memory cells (Nair et al. 2002). There are a number of Web-based tools are available for the prediction of B-cell epitope which are hosted by IEDB-AR. For the B-cell epitope prediction with high accuracy, multiple tools, including the Emini surface accessibility prediction (Emini et al. 1985), Kolaskar and Tongaonkar antigenicity scale (Kolaskar and Tongaonkar 1990), Parker hydrophilicity prediction, (Parker et al. 1986) and finally the Chou and Fasman beta turn prediction tool (Chou and Fasman 1979) were employed, because the antigenic parts of a protein belong to the beta turn regions (Rini et al. 1992).

### Homology modeling and protein variability determination of the conserved region

The structure of the conserved region was constructed by homology modelling using the MODELLER v9 (Šali et al. 1995). MODELLER is a program that implements an automated approach to comparative protein structure modelling by satisfying spatial restraints (Fiser et al. 2000, Sali and Blundell 1993). Finally, the evaluation of the predicted model was verified by using two software tools, PROCHECK (Arnold et al. 2006, Laskowski et al. 1996) and QMEAN (Benkert et al. 2011). For predicting the disorder among the amino acid sequences, DISOPRED v3 (Ward et al. 2004) server was used. In order to calculate the protein variability index the Protein variability server was implied where Wu-Kabat variability coefficient (Garcia-Boronat et al. 2008) has been used.

### Allergenicity and epitope conservancy analysis

The web-based AllerHunter server (Muh et al. 2009) was used to predict the allergenicity of our proposed epitope for vaccine development. This server predicts allergenicity through a combinational prediction, by using both integration of the Food and Agriculture Organization (FAO)/World Health Organization (WHO) allergenicity evaluation scheme and support vector machines (SVM)-pairwise sequence similarity. AllerHunter predicts allergens as well as nonallergens with high specificity. This makes AllerHunter is a very useful program for allergen cross-reactivity prediction (Liao and Noble 2003).

Epitope conservancy of the candidate epitopes was examined using a Web-based epitope conservancy tool available in IEDB analysis resource (Bui et al. 2007). The conservancy level of each potential epitope was calculated by looking for identities in all RNA-dependent RNA polymerase-L protein sequences of different strains retrieved from database.

## Results

### Analysis of the retrieved sequences with divergence and antigenicity

**Fig. 1:**
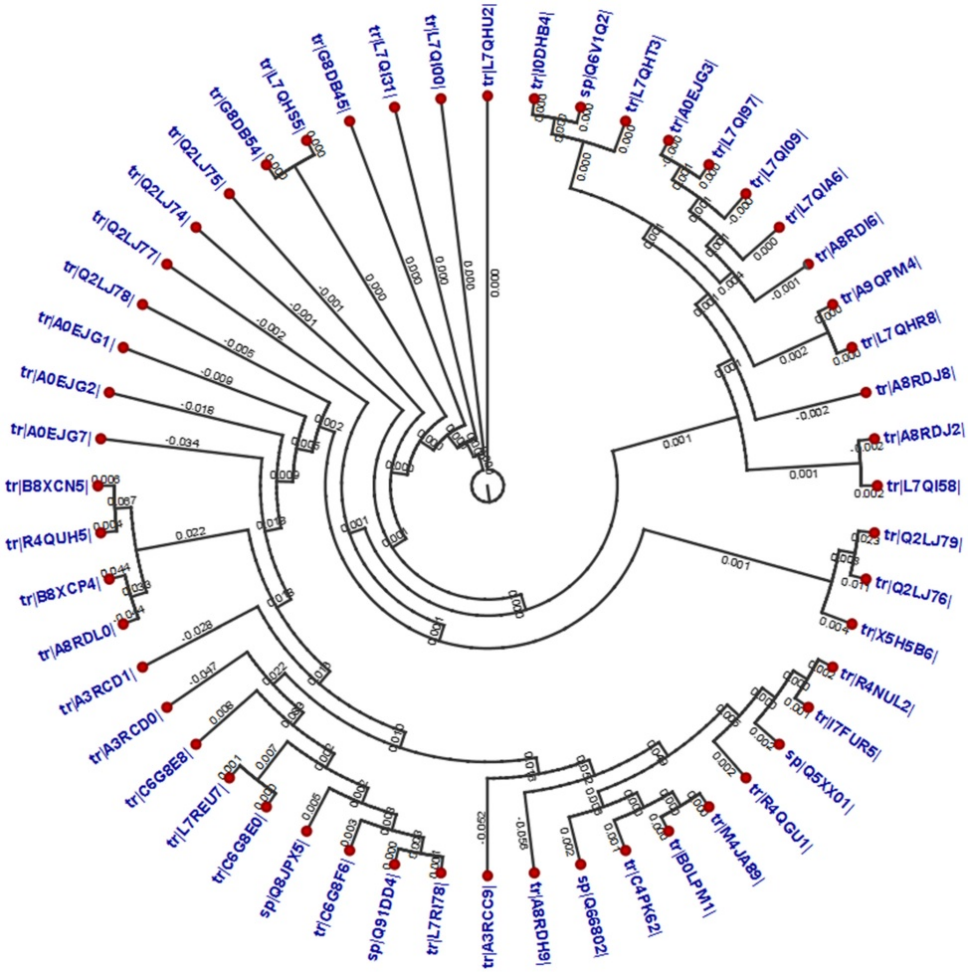
Phylogenetic tree showing the evolutionary divergence among the different RNA-dependent RNA polymerase-L proteins of the EBOV. Notes: Here, cladogram view is shown with appropriate distance among the different strains. The red dotted view indicates the node of the tree

**Fig. 2:**
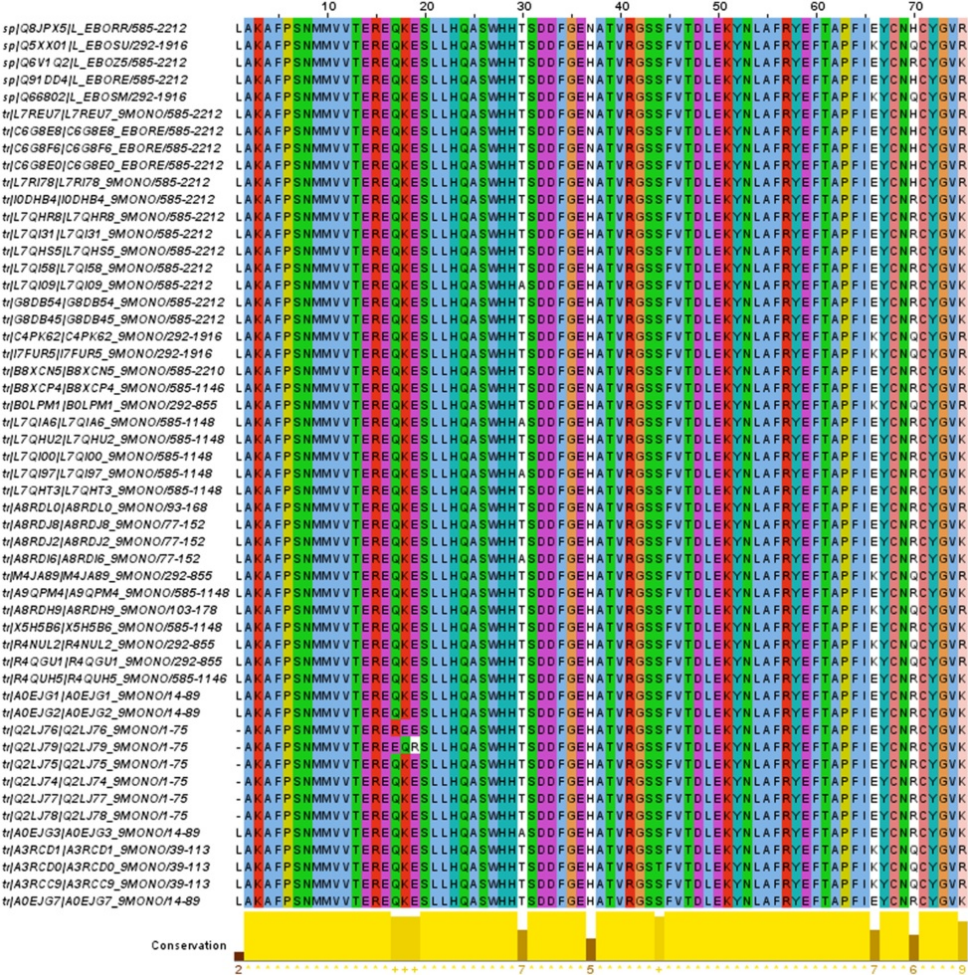
MSA of the conserved region of RNA-dependent RNA polymerase-L. Only the conserved sequences containing the proposed epitope sequence are shown here. Notes: Clustalx color is used here. Different colors indicate different amino acid residues. The yellow bas at the bottom indicates the conservation of the amino acid residues

A total of 52 RNA-dependent RNA polymerase-L protein molecules from different variants of the EBOV were retrieved from the UniProt database. The MSA of the RNA-dependent polymerase-L proteins was retrieved from BioEdit tool through ClustalW with 1000 bootstrap replicates (Additional file 1: Figure S1). CLC Sequence Viewer was used to construct phylograms from the MSA obtained from BioEdit, in order to analyze the divergence among the retrieved sequences. Phylogram of RNA-dependent RNA polymerase-L is depicted in Fig. 1. Finally, the highly conserved region from the MSA was retrieved for the further analysis. The selected conserved region is depicted in the Fig. 2, from the MSA number 586 to 660. Then the VaxiJen v2.0 server calculate the antigenicity of the conserved sequences with a score 0.4888.

### Identification of T-cell epitope and MHC interaction analysis

T-cell epitopes were selected firstly by using the NetCTL v1.2 server where the epitope prediction was confined to 12 MHC-I supertypes. Based on the combined score, the top five epitopes (Table 1) were listed for further analysis. T-cell epitopes were again predicted by the CTLPred server (Table 2). Here a combined approach of artificial neural networks and support vector machines was applied. Depending on the two analyses, the most common epitope—containing peptides, identified by both servers, was selected. The selected epitope was then used for the MHC-binding analysis.

**Table 1:** Prediction of the T-cell epitope by NetCTL server on the basis of combined score

Epitope	Start position	Combined score
FIEYCNHCY	64	2.4978
FRYEFTAPF	56	2.0697
RYEFTAPFI	57	1.6395
ESLLHQASW	19	1.2675
SFVTDLEKY	44	1.1582

**Table 2:** Prediction of the T-cell epitope by CTLPred server

Epitope	Start position	Score(ANN/SVM)
KYNLAFRYE	51	0.87/0.51591091
RYEFTAPFI	57	0.45/0.69332887
FRYEFTAPF	56	0.84/0.29033079
KAFPSNMMV	3	0.64/0.46418851
LAKAFPSNM	1	0.46/0.61842782

MHC-I-binding prediction, which was run through the Stabilized Matrix Method, predicted a wide range of *MHC-I* allele interactions for the proposed T-cell epitopes. The *MHC-I* alleles for which the epitope showed higher affinity (IC_50_ < 250 nM) are listed in Table 3. The output of the *MHC-II* interaction analysis is also shown in Table 3.

**Table 3:** MHC-I and MHC-II interaction of the proposed sequence by IEDB analysis resource

Epitope	MHC I interaction	Epitope	MHC II interaction
FRYEFTAPF	HLA-C*03:02,HLA-C*07:02, HLA-C*12:03,HLA-C*14:02, HLA-C*16:01,HLA-C*06:02, HLA-C*07:01,HLA-C*12:02, HLA-B*27:05, HLA-B*39:01	NLAFRYEFTAPFIEY	HLA-DRB3*01:01, HLA-DQA1*04:01, HLA-DRB3*02:02, HLA-DRB1*03:01, HLA-DRB1*04:01, HLA-DRB1*04:05, HLA-DRB5*01:01, HLA-DPA1*02:01, HLA-DPA1*01:03, HLA-DQA1*03:01, HLA-DRB1*07:01, HLA-DRB1*08:02, HLA-DPA1*01, HLA-DRB1*11:01, HLA-DPA1*02:01, HLA-DQA1*05:01, HLA-DPA1*02:01, HLA-DRB1*09:01, HLA-DQA1*01:01, HLA-DPA1*03:01, HLA-DRB1*15:01, HLA-DRB1*13:02, HLA-DRB1*12:01, HLA-DRB4*01:01, HLA-DQA1*05:01, HLA-DQA1*01:02

### Analysis of the population coverage

**Fig. 3:**
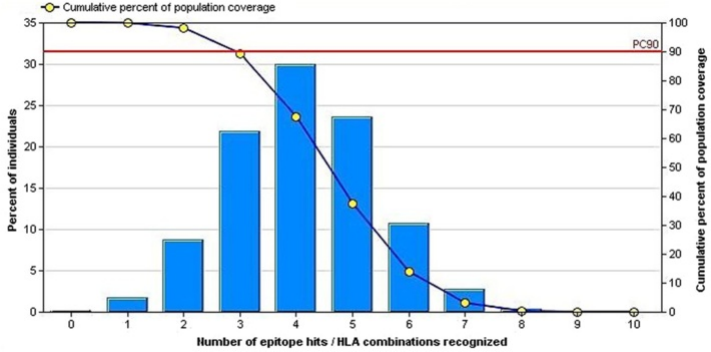
Population coverage based on MHC–I and MHC-II restriction data. The whole world populations are assessed for the proposed epitope. Notes: In the graphs, the line (−o-) represents the cumulative percentage of population coverage of the epitopes; the bars represent the population coverage for each epitope

IEDB population coverage tool analyzed the Population coverage of the proposed epitope. The combined MHC-I and MHC-II class were assessed against the whole world population with the selected *MHC-I* and *MHC-II* interacted alleles (Fig. 3).

### Prediction B-cell epitope

Here, for predicting potential B-cell epitopes, we used amino acid–based methods. According to this procedure different analysis methods were applied for the identification of a continuous B cell epitope.

**Fig. 4:**
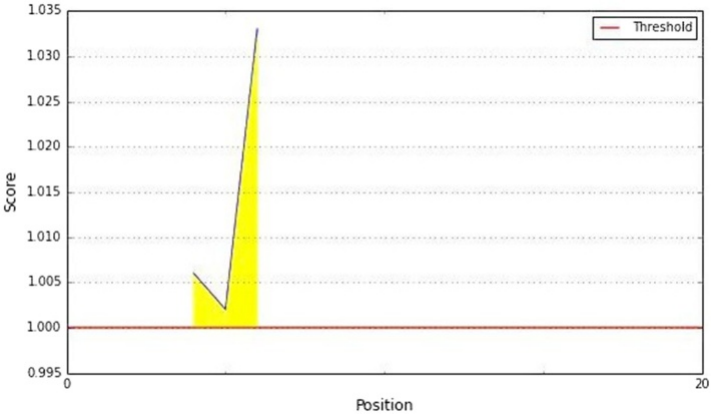
Kolaskar and Tongaonkar antigenicity prediction of the proposed epitope. Notes: The X- and Y-axes represent the sequence position and antigenic propensity score, respectively. The threshold value is 1.0. The regions above the threshold are antigenic, shown in yellow

The Kolaskar and Tongaonkar antigenicity scale was used for assessing the antigenic property of the peptides. The average antigenic propensity of the protein was 1.014, with a maximum of 1.033 and a minimum of 1.002. For the protein the antigenic determination threshold value was 1.0, where all values equal or greater than 1.0 were potential antigenic determinants. The antigenic plot is depicted in the Fig. 4.

**Fig. 5:**
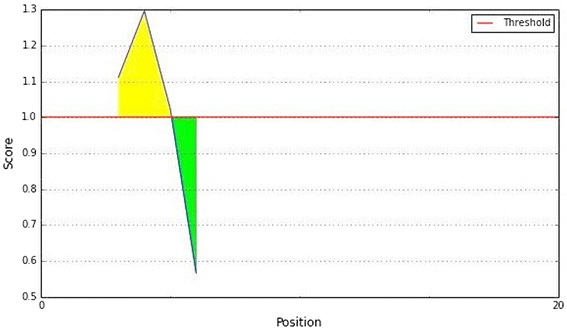
Emini surface accessibility prediction of the proposed epitope, with a minimum propensity score of 0.566 and maximum score of 1.297. Notes: The X- and Y-axes represent the sequence position and surface probability, respectively. The threshold value is 1.0. The regions above the threshold are antigenic, shown in yellow

**Fig. 6:**
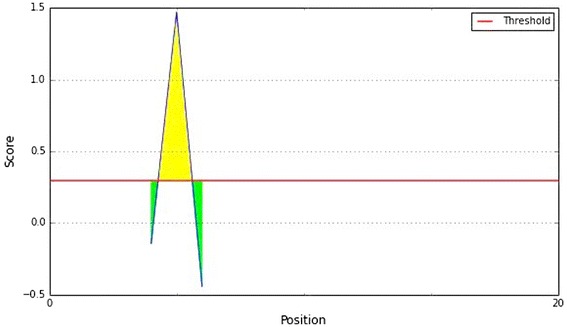
Parker hydrophilicity prediction of the epitope, with a minimum propensity score of −0.443 and maximum score of 1.471. Notes: The X- and Y-axes represent the sequence position and antigenic propensity score, respectively. The threshold value is 1.0. The regions above the threshold are antigenic, shown in yellow

**Fig. 7:**
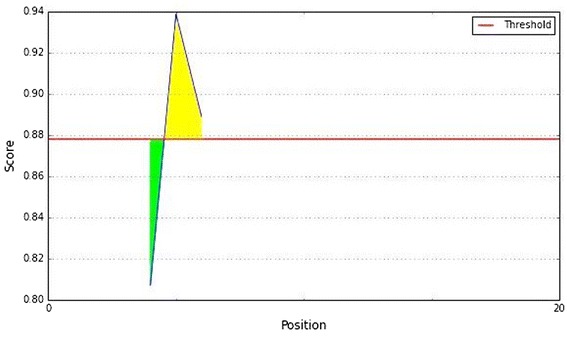
Chou and Fasman beta turn prediction of the epitope,with a minimum propensity score of 0.878 and maximum score of 0.939. Notes: The X- and Y-axes represent the sequence position and antigenic propensity score, respectively. The threshold value is 1.0. The regions above the threshold are antigenic, shown in yellow

To be a potent B cell epitope, it must be surface accessible. Hence, Emini surface accessibility prediction was employed, with a maximum propensity score of 1.297 at threshold 1.0 (Fig. 5). To strengthen our support for the prediction of the epitope to elicit B cell response the Parker hydrophilicity and the Chou and Fasman beta turn prediction were employed. Those are described in the Figs. 6 and 7.

### Structure analysis and protein variability determination

**Fig. 8:**
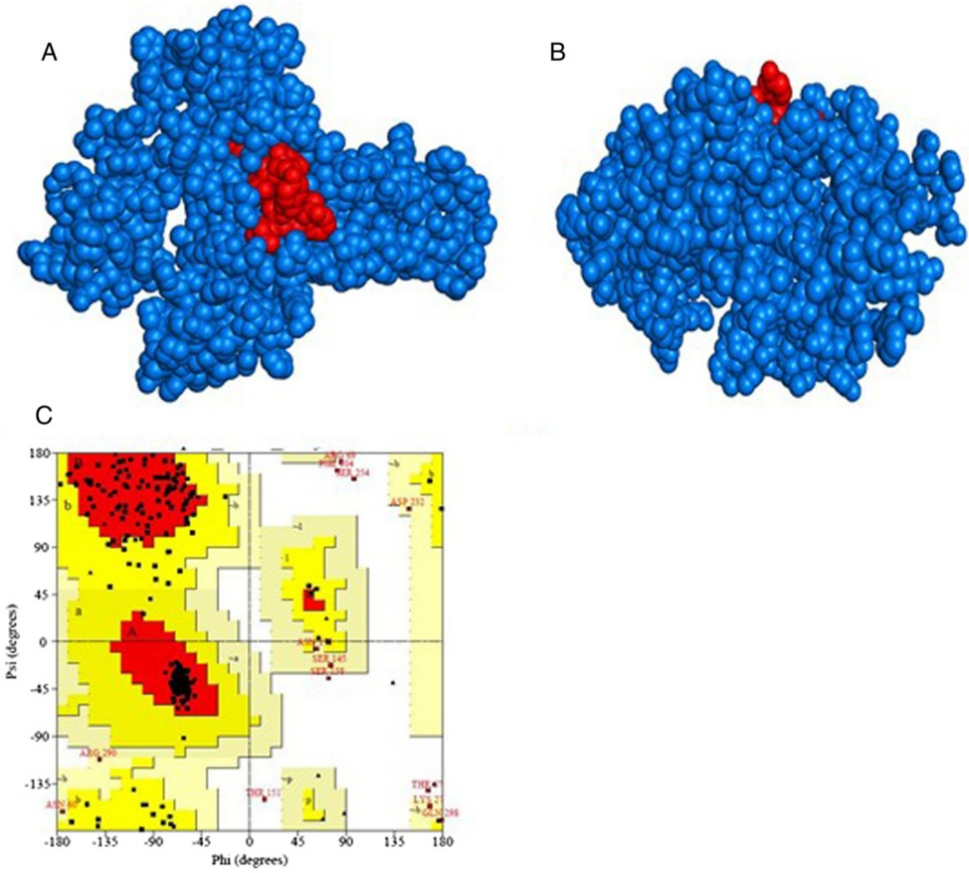
Three-dimensional model of the RNA-dependent RNA polymerase-L with the proposed epitope and validation. Notes: Two different view of the modeled protein (Blue spherical) with the predicted epitope (Red spherical). **a** Top view. **b** Side view. The outerside location of the epitope indicates its surface accessibility. **c** Ramachandran plot of the predicted model shows that most of the residues are in the allowed region of the plot, proving the validity of the model

**Fig. 9:**
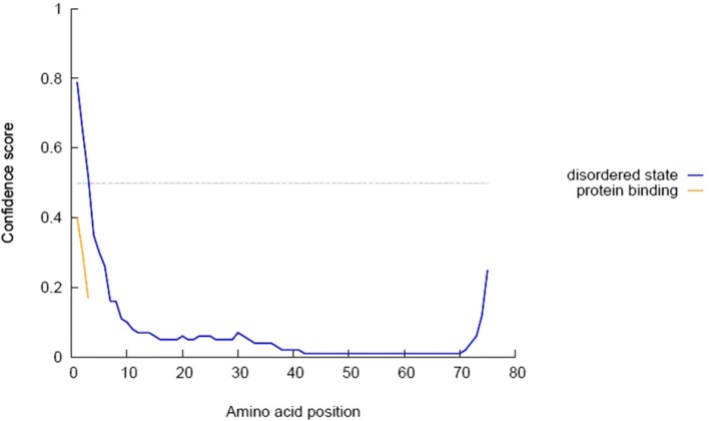
Disorder prediction of the conserved antigenic amino acid sequences. Here, our proposed epitope lies outside (56–64) of the disordered region to secure its potentiality as an effective epitope. Notes: Amino acids in the input sequence are considered disordered when the blue line is above the gray dashed line, that is, when the confidence score is 0.5. The orange line shows the confidence score of the disordered protein-binding residue predictions

**Fig. 10:**
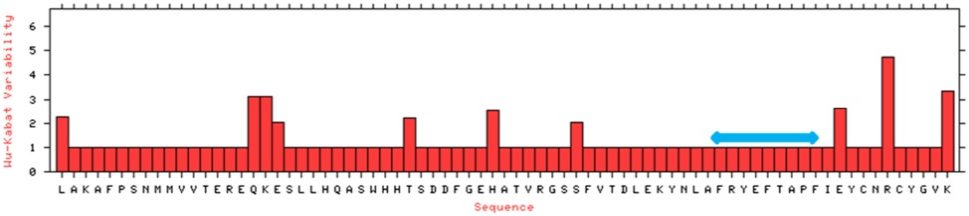
Protein variability index of the conserved peptides of all the sequences. The prediction suggests that our proposed epitope “FRYEFTAPF” falls in the invariable region (Blue line). Notes: The conservancy threshold was 1.0 in this analysis. The X-axis indicates the amino acid positions in the sequences and the Y-axis indicates the Shannon variability score

Homology model of the conserved region was obtained by the MODELLER software, which is shown in Fig. 8a and b. PROCHECK server validated the stereochemical quality of the model through Ramachandran Plot (Fig. 8c), andQMEAN server also assessed the tertiary structure, with a Qmean6 score of 0.327. DISOPRED v3 server predicted the disorder of the conserved peptide in order to get insight about the disorder among the conserved sequences, which is depicted in Fig. 9. Protein variability server predicted the variability of the conserved region of the RNA-dependent RNA polymerase-L (Fig. 10) to ensure that the proposed epitope is within the invariable region.

### Epitope conservancy and allergenicity analysis

Conservation analyses of the proposed epitopes were analyzed by the IEDB conservancy analysis tool that is shown in Table 4. AllerHunter server predicted the allergenicity of the queried epitope with a score was 0.03 (sensitivity =94.4. %, specificity =70.3 %).

**Table 4:** Epitope conservancy analysis

Peptide sequence	Peptide length	Percentage of protein sequence match	Maximum identity
FRYEFTAPF	9	100 % (52/52)	100 %
NLAFRYEFTAPFIEY	15	100 % (52/52)	100 %

## Discussion

Our world is the habitation of more than seven billion people now. With the upgrade of medical science, new viruses along with their causing diseases are also emerging. Ebola virus is such kinds of virus with a deadly outrage of their endemic nature especially in Africa in recent time (Evans and Popova 2015). Till now there is no potential treatment for this virus to combat its deadly effects.

Recent time, the immunoinformatics approach give us some sort of hope for the design of an effective therapeutics, like vaccine, in association with the advancement of sequence based technology. Similar approaches have been used successfully for identifying vaccine candidates in several pathogens viz. human corona virus (Oany et al. 2008), Saint Louis encephalitis virus (Hasan et al. 2013), Crimean–Congo hemorrhagic fever virus (Oany et al. 2015), Chikungunya virus (Hasan et al. 2015) and some others. The *in vitro* validation of this type of work has also been proven in recent time (Khan et al. 2014).

Though epitope-based vaccine designing has become a familiar approach, in the case of EBOV no significant work yet has been done. EBOV is an RNA virus which has genetic blueprints made of RNA instead of DNA. Creating vaccines is particularly difficult for RNA viruses as they can quickly mutate their different exposed proteins (Twiddy et al. 2003). Therefore the most potential way to create stable antiviral therapies against RNA viruses including EBOV is to target the transcription or replication machinery. Scientists revealed that RNA-dependent RNA polymerase-L (EBOL) is an important cellular component for the transcription and replication of the EBOV genome. When an EBOV infects a cell, its RNA genetic blueprint enters the cell along with RNA-dependent RNA polymerase-L. This polymerase normally “read” the RNA genetic blueprint in order to synthesize mRNA, which then leads to the formation of viral proteins as well as viral replication and more viral particles are produced. For these two vital involvements at the gateway, this protein was targeted to design most potential epitopes using *in silico* computational approaches.

In the current study, firstly all the available sequences of RNA-dependent RNA polymerase-Lwere retrieved from database. Then antigenicity of the conserved peptides, generated by multiple sequence alignment was predicted by VaxiJen, which suggested their ability to elicit potential immune response. Sequence based bioinformatics approaches were applied to predict both B cell and T cell epitopes for conferring immunity in different ways. Though at present, most of the vaccines are based on B cell immunity; vaccines based on T cell epitope have been encouraged recently. It is because, with time humoral response from memory B cells can be overcome easily by antigenic drift, while cell mediated immunity often provides long lasting immunity (Bacchetta et al. 2005, Igietseme et al. 2004). Cytotoxic CD8^+^T lymphocytes (CTL) inhibit the spread of infectious agents by recognizing and killing infected cells or secreting specific antiviral cytokines (Garcia et al. 1999, Shrestha and Diamond 2004). Thus, vaccination based on T cell epitope is a unique approach to obtain strong immune response against infectious agents, such as, viruses (Klein et al. 2005).

Both NetCTL and CTLPred server were used to find epitopes for the activation of T-cell immunity with potential antigenicity. By examining the output it was predicted that FRYEFTAPF would be the best epitope candidate and was further subjected for binding proficiency analysis.

Length is an important factor to consider for peptide antigen binding with MHC or TCR or both. T cell epitopes presented by MHC class I molecules are generally peptides between 8 and 11 amino acids in length. We therefore set peptide lengths at 9 before making software based MHC class I T cell epitope identification using immune epitope database (IEDB). Analysis revealed that the core epitope “FRYEFTAPF” would interact with ten different MHC class I alleles. On the other hand, the complete peptide “NLAFRYEFTAPFIEY” interacts with the highest numbers of MHC class II alleles (as many as 26 alleles).

Along with the T-cell epitope, in our study, attention was also given to the B-cell epitope, which can induce both primary and secondary humoral immunity (Trainor et al. 2007). Multiple prediction methods were applied to determine the B-cell epitope considering several criteria of antigenicity, hydrophilicity, surface accessibility, and beta-turn. Our proposed epitope has met all the criteria of the above B-cell prediction methods.

The three-dimensional model of the conserved protein ensured the exact location of the epitope outside of the protein (Fig. 8a and b) surface and the model validity was assessed by Ramachandran Plot (Fig. 8c), whereby 87.8 % amino acid residues were found within the favored region. The epitope was also treated as suitable candidate for vaccine through tenabled its position in the conserved sequence, by the Discopred and protein variability server (Figs. 9 and 10).

Conservancy is the most important criterion of an epitope to consider it for vaccine development. Conservancy analysis of our proposed epitope showed 100 % conservancy among all the available sequences. Another important feature of the peptide vaccine is its allergenicity (McKeever et al. 2004). *In silico* analysis revealed that the proposed epitope is nonallergenic in nature.

Wide range population coverage must be needed for a potential vaccine aspirant. At this point, our proposed epitope covers a remarkable population of 99.87 % for both types of *MHC allele* throughout the world population. That makes the epitope as a supreme candidate for vaccine consideration.

Finally, from the above *in silico* analysis, we are really optimistic that our proposed epitope would trigger an immune response *in vitro and in vivo*.

## Conclusion

A number of approaches exist for new vaccine development, such as recombinant vaccines, sub-unit protein and DNA vaccines, auxotrophic organisms to deliver genes and so on. Current study is an attempt to identify potential epitope targets against EBOV using different computational tools. It is quite obvious that in order to minimize the deadly effects of EBOV, highly potential drugs are immediately required and these *in silico* approaches will reduce the wet lab efforts with higher probability of success. Therefore, it is concluded that the identified epitope may be exploited further for developing epitope-based vaccine against EBOV. Nevertheless, the initial hints we obtained will help to prioritize potential therapeutics for EBOV.

## Additional file

Additional file 1: Figure S1. MSA of the RNA-dependent polymerase-L proteins of the different EBOV. (PNG 1540 kb)
